# Meteorological and surface radiation data observed at the Brazilian Antarctic station on King George Island

**DOI:** 10.1016/j.dib.2019.104245

**Published:** 2019-07-12

**Authors:** Jacyra Soares, Marco Alves, Flavia Noronha Dutra Ribeiro, Georgia Codato

**Affiliations:** University of Sao Paulo, Sao Paulo, Brazil

**Keywords:** Surface radiation balance observational data, Surface wind and temperature observational data, Antarctic peninsula, King George Island, Antarctica

## Abstract

The observational data described here was collected between 28 February 2011 and 30 November 2015. The data analysis and interpretation were published in the article “Surface radiation balance and weather conditions on a non-glaciated coastal area in the Antarctic region” [1]. An instrumented tower located on the non-glaciated coastal area of the of the Brazilian Antarctic Comandante Ferraz Station, at King George Island, Antarctic Peninsula was used. It was collected data of air temperature and relative humidity, wind speed and direction, barometric pressure, incident and reflected shortwave radiation, longwave radiation emitted by atmosphere and by surface, and net radiation with a sampling frequency of 0.1 Hz. The data was stored as 5-min averages and automatically transmitted to the Air-Sea Interaction Laboratory, at the University of São Paulo, Brazil. The dataset is hosted in the Mendeley repository.

**Table:** Specifications table

Subject area	Environmental Science
More specific subject area	Meteorology
Type of data	Table
How data was acquired	All variables were measured in situ on an instrumented tower. Air temperature and relative humidity were measured using a CS215 Campbell Sci. Inc. at 2.2 m of height; wind speed and direction with a 05103, R. M. Young Company at 10.6 m of height; barometric pressure using a CS106 Vaisala at 1.5 m of height and the radiation balance components using a CNR4 + ventilation unit CVF4 Kipp Zonen at 3.4 m of height. The net radiation measurements were obtained during the first period of the project utilizing a CNR4 + ventilation unit CVF4 Kipp Zonen, and during the last period a NR Lite 2, Kipp Zonen always at 3.4 m of height.
Data format	Raw. Only data with values below or above the specific limit values of each variable were taken from the data set.
Experimental factors	The meteorological tower is within a distance of 70 m from the Martel Inlet by the eastern side. At the south sector the Admiralty Bay is found. The Flagstaff Hill, with a maximum height of 267 m and with its base about 400 m distant from the tower is located at the western sector.
Experimental features	It was collected data of air temperature and relative humidity, wind speed and direction, barometric pressure, incident and reflected shortwave radiation, longwave radiation emitted by atmosphere and by surface, and net radiation with a sampling frequency of 0.1 Hz. The data was stored as 5-min averages and automatically transmitted to the Air-Sea Interaction Laboratory, at the University of São Paulo, Brazil.
Data source location	Measurements were performed by the ETA Project (acronym for the original name in Portuguese, Study of Turbulence in Antarctica), at Brazilian Antarctic Comandante Ferraz Station (62°05′07″ S, 58°23′33″ W), King George Island, Antarctic Peninsula.
Data accessibility	https://data.mendeley.com/datasets/gydv43hcxy/2 (https://doi.org/10.17632/gydv43hcxy.2)
Related research article	J. Soares, M. Alves, F.N.D. Ribeiro, G. Codato, Surface radiation balance and weather conditions on a non-glaciated coastal area in the Antarctic region, Polar Sci. https://doi.org/10.1016/j.polar.2019.04.001.

**Table:** 

**Value of the data** Extreme weather conditions in Antarctica make it one of the most challenging ecosystems on Earth, with great difficulty in obtaining meteorological data. The scarcity of in situ climatic records makes it important to gather as much data as possible from all existing sources to understand the recent climate changes, that appear to be occurring in this important region, and to advance the understanding of the numerical results obtained by global and regional atmospheric and oceanic models representing Antarctica. Ferraz Station is located in a non-glacial coastal area of King George Island, characterized by a complex topography, which further complicates the collection of data. There are very few publications involving in situ surface meteorological measurements obtained at Ferraz Station and virtually nothing is known about the radiation balance at this site. This lack of observational knowledge makes the dataset presented here valuable. In summary. • Observational data from the surface radiation balance, together with meteorological parameters, such as the dataset presented here, are important for diagnostic and prognostic studies of climate change and for environmental monitoring. • Environmental numeric models use parametrizations that need to be pre-calibrated using observed data, such as the dataset shown here. • Biological studies carried out at the Ferraz station depend on the knowledge of the local surface meteorological variables.

## 1 Data

**Fig. 1:**
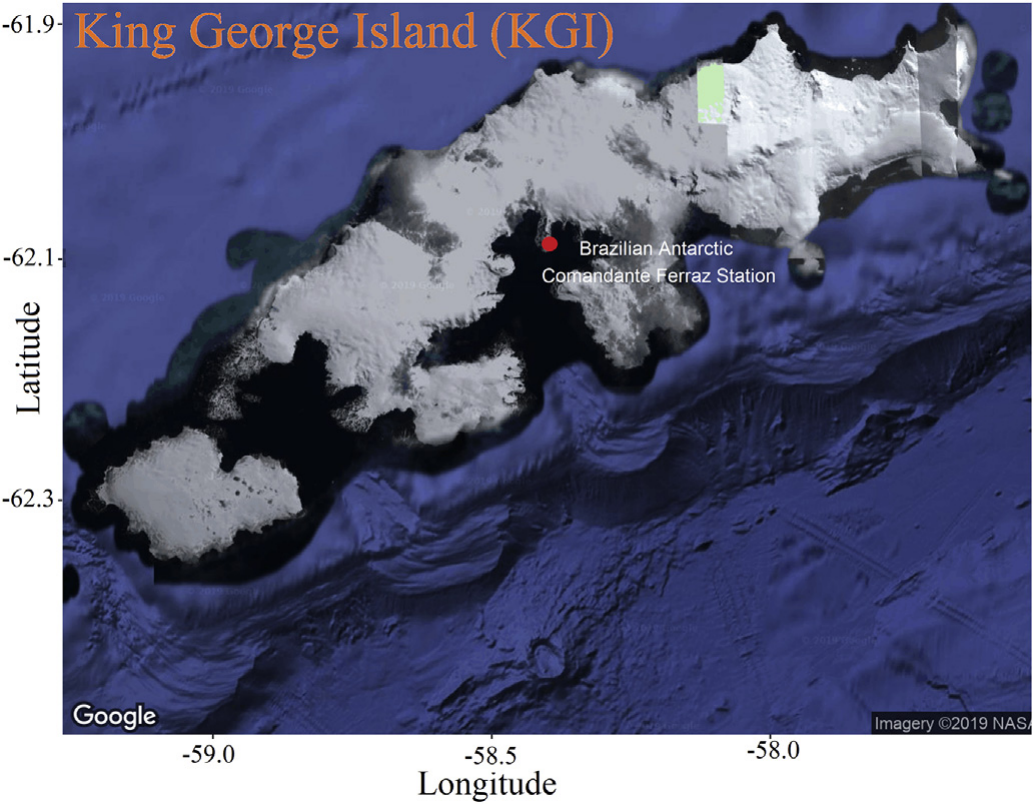
Location of the Brazilian Antarctic Comandante Ferraz Station (red dot) on King George Island. Image from Google.

**Fig. 2:**
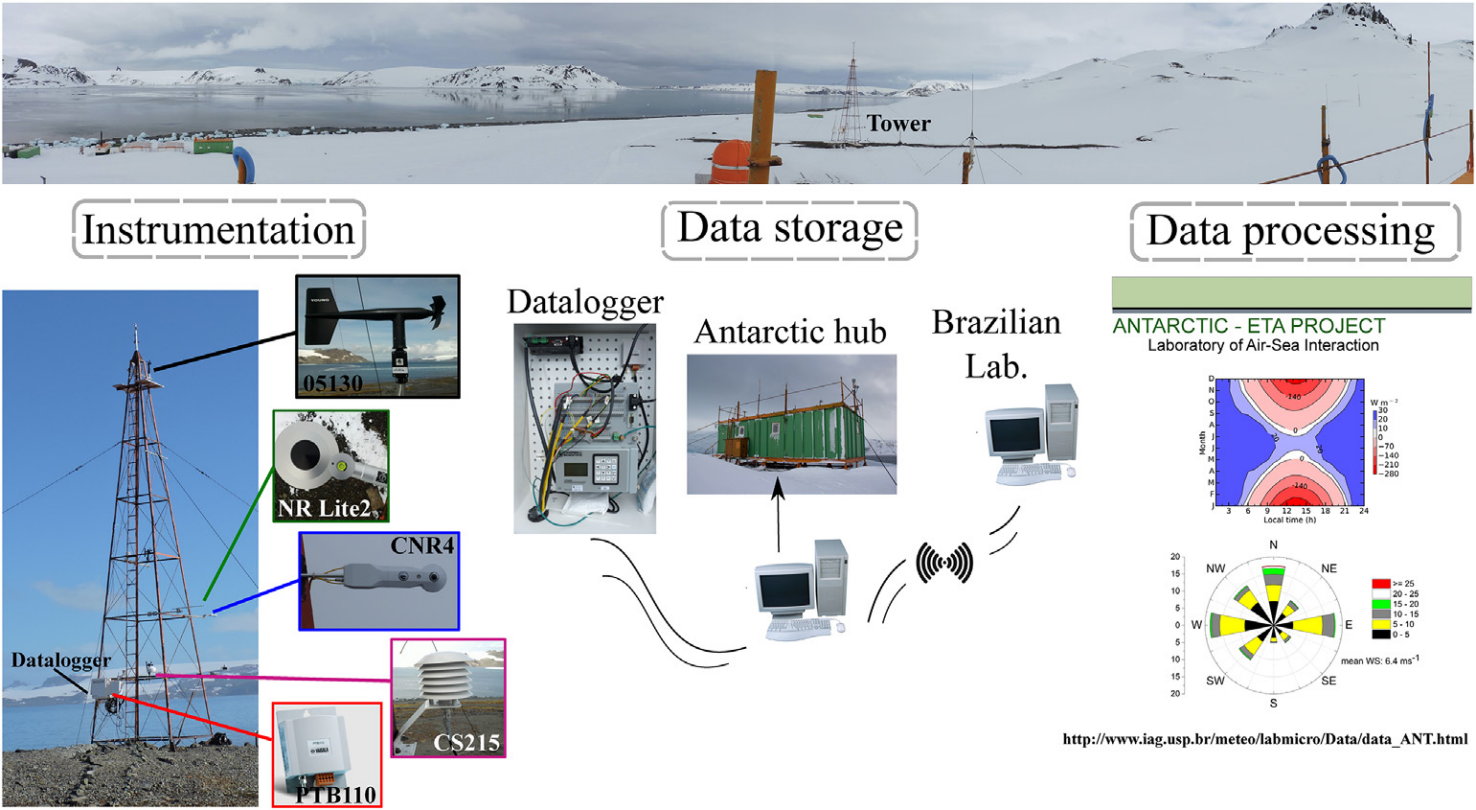
Scheme for the acquisition, storage and transmission of ETA Project data.

The data was collected at Brazilian Antarctic Comandante Ferraz Station (62°05′07″ S, 58°23′33″ W), at King George Island (Fig. 1), Antarctic Peninsula. The observed data (air temperature, air relative humidity, wind speed and direction, barometric pressure, incident and reflected shortwave radiation, longwave radiation emitted by atmosphere and by surface, and net radiation) was gathered between 28 February 2011 and 30 November 2015 with some smalls and one large time interruption (Table 1), using an instrumented tower, as displayed in Fig. 2. The data, with sampling frequency of 0.1 Hz, was stored as 5-min average by a datalogger. The 5-min average data is hosted in the Mendeley repository [2].

**Table 1:** Equipment installed in Ferraz Station (KGI) during the ETA project.

Variable (symbol)	Time period	Sensor	Height (m)
Air temperature	21 Nov 2013–10 Oct 2014 01 Nov 2014–30 Nov 2015	CS215 Campbell Sci. Inc.	2.2
Air relative humidity (RH)			
Wind speed (WS)	21 Nov 2013–10 Oct 2014 26 Oct 2014–03 Oct 2015 23 Nov 2015–30 Nov 2015	05103 R. M. Young Company	10.6
Wind direction (WD)	11 Nov2013–03 Oct 2014 21 Oct 2014–18 Jul - 15 23 Nov 2015–30 Nov 15		
Barometric pressure (PRE)	28 Feb 2011–24 Feb 2012 21 Nov 2013–10 Oct 2014 02 Nov 2014–10 Nov 2014 21 Nov 2014–30 Nov 2015	PT110 Vaisala	1.5
Incident shortwave radiation (SW↓)	28 Feb 2011–24 Feb 2012 21 Mar 2014–21 Nov 2015	CNR4 + ventilation unit CVF4 Kipp Zonen	3.4
Reflected shortwave radiation (SW↑)			
Longwave radiation emitted by atmosphere (LW↓)	28 Feb 2011–24 Feb 2012		
Longwave radiation emitted by surface (LW↑)			
Net radiation (Rn)	28 Feb 2011–24 Feb 2012	CNR4 + ventilation unit CVF4 Kipp Zonen	
	12 Nov 2013–11 Oct 2014 22 Nov 2014–21 Nov 2015	NR Lite 2 Kipp Zonen

## 2 Experimental design, materials, and methods

The extreme weather conditions prevailing at Brazilian Station, along with its special location in a non-glacial coastal area of King George Island (Fig. 1), characterized by complex topography and land cover continuously affected by the temporal and spatial distribution of ice/snow makes Ferraz Station a challenging place for observational studies of surface meteorological conditions.

The atmospheric dataset was obtained using a 12-m tower located in a coastal area of Brazilian Station, 70 m from the Martel Inlet on the east side. In the northern sector is found the Admiralty Bay and Stenhouse Glacier. Flagstaff Hill, with a maximum height of 267 m and its base about 400 m away from the tower, is located in the western sector. The Admiralty Bay is also present in the southern sector [1].

Without snow, the surface where the tower is placed consists of rocks and gravels. Near the tower (<10 m) there is a shallow lake (South Lake), which is often frozen except for some summer days.

The ETA Project was carried out between 28 February 2011 and 30 November 2015. The data was obtained with a sampling frequency of 0.1 Hz and stored as 5-min averages by a CR5000 datalogger (Campbell Scientific Inc., UK). The data was automatically transmitted to the Air-Sea Interaction Laboratory, at the University of São Paulo, Brazil, as summarized in Fig. 2. Differences in measurement lengths depend on installation date of equipment and on technical problems [1]. The equipment and their respective heights in the tower are shown in Table 1.

The local time (LT) was used as the standard time (LT = UTC - 4). The duration of the day, in Ferraz Station, varies from 05 hours to 07 minutes on June 19 to 19 hours and 47 minutes on December 20.

The radiation measurements in the Antarctic region may have some problems due to the effects of icing, tilted sensor, and poor cosine response [3], [4]. Ice deposition on the sensors was avoided using sensor ventilation and heating. The horizontality of the sensors was periodically verified and adjusted when needed. To weak the effects associated with the poor cosine response from the shortwave sensor present in a shorter temporal resolution data it is recommended to use daily accumulated values of shortwave.
